# Food groups and risk of squamous cell carcinoma of the oesophagus: a case–control study in Uruguay

**DOI:** 10.1038/sj.bjc.6601239

**Published:** 2003-09-30

**Authors:** E De Stefani, H Deneo-Pellegrini, A L Ronco, P Boffetta, P Brennan, N Muñoz, X Castellsagué, P Correa, M Mendilaharsu

**Affiliations:** 1 Registro Nacional de Cáncer, Montevideo, Uruguay; 2 Unit of Environmental Cancer Epidemiology, International Agency for Research on Cancer, Lyon, France; 3 Unit of Field and Intervention Studies, International Agency for Research on Cancer, Lyon, France; 4 Servei d'Epidemiologia i Registre del Cáncer, Institut Català d?Oncologia, L'Hospitalet de Llobregat, Barcelona, Spain; 5 Louisiana State University Health Sciences Center, Department of Pathology, New Orleans, LA, USA

**Keywords:** oesophageal cancer, fruits, vegetables, stewed meat, salted meat

## Abstract

In the time period January 1998–December 2000, a case–control study on squamous cell cancer of the oesophagus was conducted in Montevideo, Uruguay. The main objective of the study was to estimate the odds ratios (ORs) associated with main food groups. For this purpose, 166 patients afflicted with squamous cell oesophageal cancer and 664 hospitalised controls were frequency matched on age and sex. Both series of patients were administered with a structured questionnaire. Aside from queries related with tobacco smoking, alcohol drinking and maté drinking, patients were interviewed with a food-frequency questionnaire (FFQ) on 64 items, representative of the usual Uruguayan diet. Red meat, salted meat and boiled meat displayed strong direct associations (OR for red meat 2.4, 95% CI 1.4–4.2). On the other hand, fish and total white meat showed moderate protective effect (OR for total white meat 0.5, 95% CI 0.3–0.9). Total fruit intake displayed a strong inverse association (OR 0.2, 95% CI 0.1-0.4), whereas total vegetable consumption presented a weak inverse association (OR for total vegetable intake 0.7, 95% CI 0.4–1.2). These results suggest that vegetables, mainly cooked vegetables, are rich in thermolabile protective substances. On the other hand, boiled (stewed) meat, which is ingested at high temperature could be, like maté, a risk factor for squamous cell cancer of the oesophagus.

Nutritional deficiencies have been suspected as risk factors for squamous cell oesophageal cancer for more than 20 years in Eastern countries, like Iran and China (Cook-Mozaffari *et al*, 1979; Thurnham *et al*, 1988; Hu *et al*, 1994). Recent reviews reported on the protective role of vegetables and fruits (Cheng and Day 1996; World Cancer Research Fund 1997). On the other hand, tobacco smoking and alcohol drinking have been considered as the main risk factors in Western countries, like US and European countries (Pottern *et al*, 1981; Launoy *et al*, 1998; Bosetti *et al*, 2000). Even in Western countries, diet could play a major role (Cheng and Day, 1996, Launoy *et al*, 1998). South American countries display high rates of oesophageal cancer, and six case–control studies (Victora *et al*, 1987; De Stefani *et al*, 1990,^1991^; Castelletto *et al*, 1994; Rolón *et al*, 1995; Castellsagué *et al*, 2000) suggested that hot-maté drinking could be a risk factor for this malignancy. In these studies, tobacco smoking, alcohol drinking and diet also played an important role. Nevertheless, in these studies food groups were restricted by a limited food-frequency questionnaire (FFQ). Finally, two studies, conducted rather recently in Uruguay, examined in detail the role of meat and plant foods in the aetiology of oesophageal cancer (De Stefani *et al*, 1999,^2001^). Since the present study examined 64 food items, and included more than 30 new cases of the malignancy, we considered that the results could be more informative than the results of previous studies.

This study, like other hospital based case–control studies, is limited by a number of drawbacks. Recall bias is an important limitation, mainly for cases, and could result in misclassification bias. For this reason, we decided to interview patients about their consumption habits 2 years before the date of the interview. Interviewer bias was avoided by careful training of the workers who personally conducted the interviews. Finally, the lack of validation study of the FFQ is a limitation. On the other hand, the high response rates, both for cases and controls are a strength of this research. Therefore, we decided to examine the role of foods in the aetiology of squamous cell carcinoma of the oesophagus.

## MATERIAL AND METHODS

In the time period January 1998–December 2001, a case–control study on squamous cell carcinoma of the oesophagus was carried out in the four major hospitals in Montevideo, Uruguay. In this period, 175 newly diagnosed and histologically verified cases with squamous cell cancer of the oesophagus were considered eligible for the study. Nine patients (5.1%) were too ill and it was decided to exclude this subset of patients. The final number of cases was 166 (response rate 94.8%).

In the same time period and in the same hospitals, 692 patients with conditions not related with tobacco smoking, alcohol drinking and without recent changes in their diets were considered eligible for the study. From this initial number of patients, 28 refused the interview, leading a final total of 664 controls (response rate 95.9%). These controls were frequency matched to cases on age (in decennia) and sex. Controls were afflicted by the following diseases: eye disorders (181 patients, 27.3%), abdominal hernia (145, 21.8%), acute appendicitis (54, 8.1%), diseases of the skin (54, 8.1%), injuries (51, 7.7%), varicose veins (43, 6.5%), urinary stones (33, 5.0%), hydatid cyst (33, 5.0%) blood disorders (33, 5.0%), fractures (32, 4.8%) and osteoarticular disorders (five, 0.7%).

Both cases and controls were administered with a questionnaire shortly after admission. The interviewers were two trained social workers, mostly unawere of the dietary risk factors for oesophageal cancer. The questionnaire included the following sections: (1) a sociodemographic section, (2) a complete tobacco smoking section, (3) a complete alcohol drinking section, (4) a maté section, including queries on daily amount, years of ingestion and temperature, (5) a complete occupation history, including job titles, industry and years of employment, (6) a family history of cancer in first-degree relatives, (7) self-reported height and weight 5 years before the date of the inteview, (8) menstrual and reproductive events and (9) a FFQ on 64 food items, considered representative of the usual Uruguayan diet. This FFQ was not validated but was tested for reproducibility with reasonably good results. In brief, foods and beverages were recorded as servings per year. Foods and food groups are shown in appendix.

### Statistical methods

Food groups were categorised in quartiles, following the distribution of the control series. In order to compensate overeating or undereating, foods were energy adjusted by the residuals method of Willett and Stampfer (1986). The main food groups (meats, dairy foods, plant foods) were compared with each control condition, taking eye disorders as the referent disease by polytomous regression (Hosmer and Lemeshow, 1989). The results were homogeneous for each food group (data not shown). Relative risks, approximated by the odds ratios (ORs), were estimated by multiple logistic unconditional regression (Breslow and Day, 1980). Tests for trend were calculated after entering the categorical variables as continuous. All calculations were performed with the STATA programme (1999).

## RESULTS

The distribution of cases and controls by sociodemographic variables and selected risk factors is shown in Table 1

**Table 1:** Distribution of cases and controls by sociodemographic variables and other risk factors

**Variable**	**Cases**	**Controls**	**OR**	**95 % CI**	***P*-value for trend**
*Age (years)*					
40–49	10 (6.0)	40 (6.0)			
50–59	34 (20.5)	136 (20.5)			
60–69	60 (36.1)	240 (36.1)			
70–79	48 (28.9)	192 (28.9)			
80–89	14 (8.4)	56 (8.4)	NA (2)		
					
*Sex*					
Male	137 (82.5)	548 (82.5)			
Female	29 (17.5)	116 (17.5)	NA (2)		
*Residence*					
Montevideo	63 (37.9)	269 (40.5)	1.0	—	—
Other counties	103 (62.1)	395 (59.5)	1.1	0.8–1.6	
					
*Urban/rural status*					
Urban	123 (74.1)	501 (75.5)	1.0		
Rural	43 (25.9)	163 (24.5)	1.1	0.7–1.6	
					
*Education (years)*					
0–2	60 (36.1)	205 (30.9)	1.0		
3–5	69 (41.6)	224 (33.7)	1.0	0.7–1.6	
6+	37 (22.3)	235 (35.4)	0.5	0.3–0.8	0.008
					
*Income (US dollars)*					
⩽153	51 (30.7)	257 (38.7)	1.0		
154+	64 (38.6)	243 (36.6)	1.3	0.9–2.0	
Unknown	51 (30.7)	164 (24.7)	—	—	
					
*Body mass index*					
⩽23.2	64 (38.6)	167 (25.1)	1.0		
23.3–25.2	39 (23.5)	165 (24.9)	0.6	0.4–0.9	
25.3–27.4	30 (18.1)	166 (25.0)	0.5	0.3–0.8	
27.5+	33 (19.9)	166 (25.0)	0.5	0.3–0.8	0.002
					
*Total energy intake*					
⩽1781	35 (21.1)	166 (25.0)	1.0		
1782–2155	39 (23.5)	166 (25.0)	1.1	0.7–1.8	
2156–2585	43 (25.9)	166 (25.0)	1.2	0.7–2.0	
2586+	49 (29.5)	166 (25.0)	1.4	0.9–2.3	0.15
					
*Tobacco smoking*^c^					
Non-smokers	29 (17.5)	201 (30.3)	1.0		
1–24	19 (11.4)	134 (20.2)	0.9	0.5–1.8	
25–44	28 (16.9)	120 (18.1)	1.6	0.9–2.8	
45–64	40 (24.1)	109 (16.4)	2.5	1.5–4.4	
65+	50 (30.1)	100 (15.1)	3.5	2.0–3.9	<0.001
					
*Alcohol drinking*^d^					
Non-drinkers	50 (30.1)	273 (41.1)	1.0		
1–60	29 (17.5)	134 (20.2)	1.2	0.7–1.9	
61–120	34 (20.5)	114 (17.2)	1.6	0.9–2.6	
121–240	30 (18.1)	89 (13.4)	1.8	1.1–3.1	
241+	23 (13.9)	54 (8.1)	2.3	1.3–4.1	0.0005
					
*Maté drinking*^e^					
Non-drinkers	6 (3.6)	83 (12.5)	1.0		
0.1–0.9	36 (21.7)	126 (19.0)	3.9	1.6–9.9	
1.0–1.9	92 (55.4)	329 (49.5)	3.9	1.6–9.2	
2.0+	32 (19.3)	126 (19.0)	3.5	1.4–8.9	0.04
					
*Maté temperature*					
Warm	9 (5.7)	29 (7.6)	1.0		
Hot	117 (74.0)	324 (85.3)	1.2	0.5–2.5	
Very hot	32 (20.3)	27 (7.1)	3.8	1.5–9.9	0.0002
					
*Type of tobacco*					
Non-smokers	29 (17.5)	201 (30.3)	1.0		
Blond	85 (51.2)	320 (48.2)	1.8	1.2–2.9	
Black	52 (31.3)	143 (21.5)	2.5	1.5–4.2	0.0003
					
*Type of cigarette*					
Non-smokers	29 (17.5)	201 (30.3)	1.0		
Manufactured	34 (20.5)	149 (22.4)	1.6	0.9–2.7	
Hand-rolled	103 (62.0)	314 (47.3)	2.3	1.4–3.6	0.0002
No. of patients	166 (100)	664 (100)			

a Unadjusted odds ratios.

b Percentages among brackets.

c Pack-years.

d Mililitres of ethanol.

e Litres day^−1^.

. As expected from the frequency-matched design, age and sex were similarly distributed among both series. Also, residence and urban/rural status were mostly similar among cases and controls. Cases were significantly less educated and showed similar income compared with controls. On the other hand, cases displayed a significantly lower body mass index (*P*=0.005), whereas total calory intake was slightly higher among cases as compared with controls (*P*=0.56). Cases consumed a higher amount of cigarettes than controls (*P*<0.001), and ingested more alcohol (*P*=0.02). Maté ingestion was higher among cases compared with controls (*P*=0.01) and maté drinked very hot was associated with a strong elevation in risk (OR 3.82, 95% CI 1.47–9.93, *P*-value <0.001).

Odds ratios of squamous cell cancer of the oesophagus for significant foods are shown in Table 2

**Table 2:** Odds ratios of squamous cell cancer of the esophagus for significant food groups

**Food^a^**	**Cases^b^**	**Controls^b^**	**OR^c^**	**OR^d^**	**95% CI**
*Red meat*					
⩽234	26 (15.7)	166 (25.0)	1.0	1.0	
235–365	39 (23.5)	166 (25.0)	1.50	1.35	0.75–2.42
366–469	36 (21.5)	167 (25.1)	1.38	1.17	0.64–2.11
470+	65 (39.2)	165 (24.9)	2.51	2.43	1.40–4.23
		*P*-value trend	<0.001	0.002	
					
*White meat*					
⩽24	66 (39.8)	166 (25.0)	1.0	1.0	
25–76	37 (22.3)	166 (25.0)	0.56	0.61	0.38–1.00
77–130	32 (19.3)	166 (25.0)	0.48	0.55	0.33–0.92
131+	31 (18.7)	166 (25.0)	0.47	0.51	0.30–0.86
		*P*-value trend	0.001	0.008	
					
*Fish*					
0	56 (33.7)	166 (25.0)	1.0	1.0	
1–12	46 (27.7)	166 (25.0)	0.82	0.90	0.55–1.48
13–52	37 (22.3)	166 (25.0)	0.66	0.78	0.47–1.30
53+	27 (16.3)	166 (25.0)	0.43	0.53	0.30–0.92
		*P*-value trend	0.003	0.02	
					
*Salted meat*					
0	128 (77.1)	558 (84.0)	1.0	1.0	
1–78	14 (8.4)	66 (9.9)	0.92	0.88	0.46–1.68
79+	24 (14.5)	40 (6.0)	2.61	2.19	1.21–3.98
		*P*-value trend	0.002	0.03	
					
*Stewed meat*					
⩽78	26 (15.7)	166 (25.0)	1.0	1.0	
79–142	30 (18.1)	166 (25.0)	1.15	1.00	0.54–1.85
143–234	51 (30.7)	166 (25.0)	1.96	1.80	1.02–3.16
235+	59 (35.5)	166 (25.0)	2.27	2.04	1.16–3.58
		*P*-value trend	<0.001	0.002	
					
*Grains*					
⩽794	31 (18.7)	166 (25.0)	1.0	1.0	
795–1094	40 (24.1)	166 (25.0)	1.29	1.26	0.72–2.22
1095–1678	41 (24.7)	166 (25.0)	1.32	1.19	0.69–2.07
1679+	54 (32.5)	166 (25.0)	1.74	1.80	1.05–3.08
		*P*-value trend	0.03	0.04	
					
*Cheese*					
⩽52	54 (32.5)	166 (25.0)	1.0	1.0	
53–78	45 (27.1)	166 (25.0)	0.83	0.86	0.53–1.39
79–234	32 (19.3)	166 (25.0)	0.59	0.61	0.36–1.02
235+	35 (21.1)	166 (25.0)	0.65	0.61	0.36–1.03
		*P*-value trend	0.03	0.03	
					
*Raw vegetables*					
⩽72	65 (39.2)	166 (25.0)	1.0	1.0	
73–156	46 (27.7)	166 (25.0)	0.71	0.80	0.50–1.28
157–312	32 (19.3)	166 (25.0)	0.49	0.51	0.31–0.85
313+	23 (13.8)	166 (25.0)	0.36	0.39	0.22–0.68
		*P*-value trend	<0.001	<0.001	
					
*Citrus fruits*					
0	70 (42.2)	166 (25.0)	1.0	1.0	
1–78	48 (28.9)	166 (25.0)	0.68	0.62	0.39–0.99
79–182	35 (21.1)	166 (25.0)	0.50	0.47	0.28–0.77
183+	13 (7.8)	166 (25.0)	0.18	0.18	0.09–0.36
		p-value trend	<0.001	<0.001	
					
*Other fruits*					
⩽140	63 (37.9)	166 (25.0)	1.0	1.0	
141–262	48 (28.9)	166 (25.0)	0.76	0.80	0.50–1.27
263–488	30 (18.1)	166 (25.0)	0.48	0.54	0.32–0.91
489+	25 (15.1)	166 (25.0)	0.40	0.54	0.31–0.95
		*P*-value trend	<0.001	0.009	
					
*Total fruits*					
⩽222	78 (47.0)	166 (25.0)	1.0	1.0	
223–400	44 (26.5)	166 (25.0)	0.56	0.56	0.35–0.89
401–615	31 (18.7)	166 (25.0)	0.39	0.45	0.27–0.75
616+	13 (7.8)	166 (25.0)	0.17	0.19	0.10–0.37
		*P*-value trend	<0.001	<0.001	
					
*Total veg.and fruits*					
⩽658	67 (40.4)	166 (25.0)	1.0	1.0	
659–986	47 (28.3)	166 (25.0)	0.70	0.75	0.47–1.20
987–1405	31 (18.7)	166 (25.0)	0.46	0.53	0.32–0.89
1406+	21 (12.6)	166 (25.0)	0.31	0.38	0.21–0.68
		*P*-value trend	<0.001	<0.001	
					
*Pulses*					
⩽6	47 (28.3)	166 (25.0)	1.0	1.0	
7–17	48 (28.9)	166 (25.0)	1.02	0.75	0.45–1.24
18–36	35 (21.1)	166 (25.0)	0.74	0.66	0.39–1.12
37+	36 (21.7)	166 (25.0)	0.76	0.59	0.35–0.99
		*P*-value trend	0.15	0.04	

a Servings per year.

b Percentages among brackets.

c Unadjusted ORs.

d Odds ratios adjusted for age, sex, residence, urban/rural status, education, body mass index, tobacco smoking, alcohol drinking, maté drinking and total energy intake.

. Red meat, stewed meat, salted meat and total grains were directly associated with risk of oesophageal cancer (OR for high intake of stewed meat 2.04, 95% CI 1.16–3.58, *P*-value for trend=0.002). White meat, fish, cheese, raw vegetables, citrus fruits, other fruits, total fruits, total vegetables and fruits and pulses were inversely associated with risk of squamous cell carcinoma of the oesophagus (OR for high intake of citrus fruits 0.18, 95% CI 0.09–0.36, *P*-value for trend <0.001).

Total processed meat (see appendix), fried meat, barbecued meat, total dairy foods, butter, whole milk, ice cream, boiled eggs, fried eggs, all desserts, total fat-rich foods (red meat, processed meat, dairy foods, eggs and desserts), cooked vegetables, tubers and total plant foods (all vegetables, all fruits, grains, tubers and pulses) were not associated with risk of squamous cell carcinoma of the oesophagus.

The final model after including all significant foods is shown in Table 3

**Table 3:** Final model including food groups

**Food^a^**	**Cases^b^**	**Controls^b^**	**OR^c^**	**OR^d^**	**95 % CI**
*White meat*					
⩽24	66 (39.8)	166 (25.0)	1.0	1.0	
25–76	37 (22.3)	166 (25.0)	0.56	0.64	0.38–1.09
77–130	32 (19.3)	166 (25.0)	0.48	0.56	0.32–0.97
131+	31 (18.7)	166 (25.0)	0.47	0.54	0.31–0.96
		*P*-value trend	0.001	0.04	
					
*Salted meat*					
0	128 (77.1)	558 (84.0)	1.0	1.0	
1–78	14 (8.4)	66 (9.9)	0.92	1.00	0.50–2.01
79+	24 (14.5)	40 (6.0)	2.61	2.34	1.22–4.49
		*P*-value trend	0.001	0.04	
					
*Stewed meat*					
⩽78	26 (15.7)	166 (25.0)	1.0	1.0	
79–142	30 (18.1)	166 (25.0)	1.15	0.83	0.44–1.55
143–234	51 (30.7)	166 (25.0)	1.96	1.49	0.83–2.67
235+	59 (35.5)	166 (25.0)	2.27	1.80	1.01–3.24
		*P*-value trend	<0.001	0.007	
					
*Raw vegetables*					
⩽72	65 (39.2)	166 (25.0)	1.0	1.0	
73–156	46 (27.7)	166 (25.0)	0.71	0.99	0.61–1.64
157–312	32 (19.3)	166 (25.0)	0.49	0.65	0.38–1.10
313+	23 (13.8)	166 (25.0)	0.36	0.51	0.28–0.93
		*P*-value trend	<0.001	0.009	
					
*Citrus fruits*					
0	70 (42.2)	166 (25.0)	1.0	1.0	
1–78	48 (28.9)	166 (25.0)	0.68	0.61	0.37–0.98
79–182	35 (21.1)	166 (25.0)	0.50	0.49	0.29–0.82
183+	13 (7.8)	166 (25.0)	0.18	0.19	0.10–0.38
		*P*-value trend	<0.001	<0.001	

a Servings per year.

b Percentages among brackets.

c Unadjusted ORs.

d Odds ratios adjusted for age, sex, residence, urban/rural status, education, body mass index, tobacco smoking, alcohol drinking, maté drinking, total energy intake and for each other.

. This model included total white meat (poultry, fish), stewed meat, salted meat, raw vegetables and citrus fruits, citrus fruits being the strongest term (OR for low intake of citrus fruits 5.26, *P*-value <0.001). Among nondietary factors, tobacco smoking remained significantly associated with risk of squamous cell carcinoma of the oesophagus (OR for heavy smokers 3.45, 95% CI 1.71–7.00, *P*-value for trend <0.0001). Also, maté temperature was significantly and positively associated with the malignancy (OR for drinkers of very hot maté 2.09, 95% CI 1.25–3.53). Finally, alcohol drinking displayed a significant increased risk (OR 2.32, 95% CI 1.13–4.77) (data not included in Table 3).

## DISCUSSION

According to the results of the present study, red meat, stewed meat and salted meat were associated with moderate to strong effects on the risk of squamous cell oesophageal carcinoma. On the other hand, white meat, poultry and fish displayed moderate inverse associations with oesophageal cancer. Among plant foods, total vegetables were weakly associated with this disease, whereas raw vegetables were strongly protective. Finally, total fruits, citrus fruits and other fruits were the most protective food groups with reductions in risk close to 80%.

Previous studies on foods and risk of squamous cell oesophageal cancer (Victora *et al*, 1987; Brown *et al*, 1998; Bosetti *et al*, 2000; Castellsagué *et al*, 2000) displayed stronger effects of fruits compared with vegetables. It is possible that cooked vegetables are not associated with squamous cell of the oesophagus due to temperature of the water. More specifically, vegetables are rich in thermolabile nutrients that are destroyed by the heat in the process of cooking (Tavani *et al*, 1994; Bosetti *et al*, 2000). On the other hand, fruits are more often eaten raw, leaving nutrients not affected by the cooking process. Citrus fruits are rich in vitamin C, a potent anticarcinogen in oesophageal cancer (Gao *et al*, 1994; Bosetti *et al*, 2000; Castellsagué *et al*, 2000). Finally, the effect of fruits on previous studies was similar in both histologies, that is, squamous cell carcinoma and adenocarcinoma of the oesophagus (Tzonou *et al*, 1996; Cheng *et al*, 2000; Sharp *et al*, 2001). It should be noted that the last studies (Cheng *et al*, 2000; Sharp *et al*, 2001) were conducted only among women.

Stewed meat is a frequent food item in the Uruguayan population, particularly in people of the lower socioeconomic strata and among rural dwellers. In the present study, stewed meat was a strong risk factor for oesophageal cancer. We would suggest that stewed meat acts through thermal injury, like maté drinking (De Stefani *et al*, 1990; Castellsagué *et al*, 2000). Studies conducted in other populations implicate soups (Bosetti *et al*, 2000) and other hot beverages (Hu *et al*, 1994) as risk factors for squamous cell oesophageal cancer. Also, in this study, salted meat was directly associated with an increased risk of squamous cell oesophageal cancer. Previous studies (World Cancer Research Fund 1997; De Stefani *et al*, 1999) suggested that salted meat is a rich source of nitrosamines. These substances have been implicated in oesophageal carcinogenesis (Ohshima and Bartsch 1981).

Poultry and fish were protective foods in our study. In fact, the effect of fish was stronger than the inverse association of poultry. Previous studies reported a'protective effect of fish, in particular lean fish (Launoy *et al*, 1998; Bosetti *et al*, 2000). Fish is rich in polyunsaturated fats, like olive oil (Launoy *et al*, 1998; Bosetti *et al*, 2000). These nutrients were found as inversely associated with the risk of squamous cell oesophageal cancer. The mechanism of action of poultry is less clear in this malignancy. A previous study reported a protective effect of poultry (Bosetti *et al*, 2000). A recent case–control study on breast cancer also revealed a reduction in risk for total white meat, fish and skinless chicken (Ronco *et al*, 2003). In this study, the inverse association between skinless and nonfried chicken and breast cancer was suggested as possibly due to its lesser content of fat.

Several studies on oesophageal cancer reported a final model including nondietary and dietary factors (Victora *et al*, 1987; Castelletto *et al*, 1994; Launoy *et al*, 1998; Cheng *et al*, 2000; Sharp *et al*, 2001). In our final model, which excluded nondietary factors, citrus fruits were the more significant and stronger term (*P*-value for trend <0.001), followed by stewed meat (*P*-value=0.007) and fresh vegetables (*P*-value=0.009). Tobacco smoking, alcohol drinking and maté temperature remained as significant factors but with slightly reduced risks.

In summary, this study on squamous cell carcinoma of the oesophagus replicates previous studies on the protective effects of raw vegetables, citrus fruits and noncitrus fruits. Also, salted meat, a rich source of nitrosamines, was a strong risk factor. Finally, boiled meat, a component of stews that are ingested very hot is, possibly, a risk factor for this malignancy. The effect of this type of meat is much higher among smokers of black tobacco and hand-rolled cigarettes, compared with smokers of blond tobacco and commercial cigarettes.

## APPENDIX

### FOOD ITEMS AND FOOD GROUPS

*Total meat*: beef, lamb, poultry, fish, bacon, sausage, blood pudding, liver, mortadella, salami, saucisson, hotdog, ham, salted meat.

*Red meat*: beef, lamb.

*White meat*: poultry, fish.

*Processed meat*: bacon, sausage, blood pudding, mortadella, salami, saucisson, hotdog, ham, salted meat.

*Dairy foods*: cheese, butter, whole milk, ice cream.

*Eggs*: boiled eggs, fried eggs, mayonnaise.

*Desserts*: ‘dulce de leche’ (milk with sugar), rice pudding, custard, marmalade, cake, croissant.

*Fat-rich foods*: red meat, processed meat, dairy foods, eggs, desserts.

*Grains*: white rice, maize, polenta, pasta, white bread.

*Raw vegetables*: carrot, tomato, lettuce, onion.

*Cooked vegetables*: garlic, swiss chard, spinach, beetroot, winter squash, cabbage, cauliflower, zucchini, red pepper.

*Total vegetables*: raw vegetables, cooked vegetables.

*Citrus fruits*: orange, tangerine.

*Other fruits*: apple, pear, grape, peach, plum, banana, plum, figs, fruit cocktail.

*Total fruits*: citrus fruits, other fruits.

*Total vegetables and fruits*: total vegetables, total fruits.

*Tubers*: potato, sweet potato.

*Pulses*: chickpeas, kidney bean, lentil.

*Total plant foods*: grains, total vegetables, total fruits, tubers, pulses.
